# Suicide prevention in Bangladesh: The role of family

**DOI:** 10.1002/brb3.2562

**Published:** 2022-04-10

**Authors:** S M Yasir Arafat, Tamkeen Saleem, Todd M. Edwards, Syeda Ayat‐e‐Zainab Ali, Murad M. Khan

**Affiliations:** ^1^ Department of Psychiatry Enam Medical College and Hospital Dhaka Bangladesh; ^2^ Department of Psychology International Islamic University Islamabad Pakistan; ^3^ Marital and Family Therapy Program University of San Diego San Diego California USA; ^4^ Department of Psychiatry Aga Khan University Karachi Pakistan

**Keywords:** family, prevention, risk factors, suicide and family, suicide in Bangladesh

## Abstract

**Background:**

Suicide is a public health problem that gets little attention in Bangladesh especially in prevention aspects. Recent studies revealed that a significant portion of risk factors is closely related to family events. However, potential prevention strategies considering the family structure and involving family dynamics of Bangladesh have not been discussed.

**Objectives:**

We aim to highlight areas of family vulnerability and resilience when the threat of suicide is present, as well as the potential roles of family in suicide prevention in Bangladesh.

**Methods:**

We conducted a thorough narrative and focused literature search and synthesized evidence based on available articles discussing suicidality and family dynamics in Bangladesh.

**Results:**

Risk factors for suicide prevailing in the family have been organized, and several strategies for coping with family risk factors, including marital discord and family conflict have been proposed for testing empirically.

**Conclusions:**

The family has an important role to play in suicide prevention in Bangladesh. However, potential prevention strategies and their effectiveness have been untapped in the country. Studies are warranted to test the effectiveness of the proposed strategies.

## Graphical abstract

Suicide is a public health problem that gets little attention in Bangladesh. We conducted a thorough narrative search and synthesized evidence discussing suicidality and family dynamics in Bangladesh. Risk factors for suicide prevailing in the family have been organized and several strategies for coping with family risk factors, including marital discord and family conflict have been proposed for testing empirically.

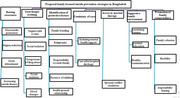

## 1 INTRODUCTION

Each year, approximately 700,000 people die by suicide, where about 77% of suicides occur in low‐ and middle‐income countries (World Health Organization [WHO], 2021). Research has focused on suicide as an individual problem; yet, there is a need to view suicide as a family experience and to use multiple perspectives for investigating the role of families in helping prevent suicide (Frey et al., 2016). Suicide can be an act of hopelessness, anger, resentment, or escape from unbearable pain and distress, which has been linked with earlier bonding conflicts within the family system, interpersonal deficits, and a lack of perceived social support (Prabhu et al., 2010). Consequently, a death by suicide has across‐the‐board effect on a large variety of people, including family members, friends, contacts, and healthcare professionals (Shear & Zisook, 2014). The suicide may have an impact on family like there may be disruption in relationship between the family members, loss of intimacy, guilt or blame among members, mental distress and other psychological problems, traumatic stress, grief, financial crisis, and a tendency to attempt suicide (Ara et al., 2016). Nevertheless, a positive milieu within the family, along with clear and transparent communication, could help in the identification of risk factors and prevent suicidal behaviors (Edwards et al., 2021). Unfortunately, there is limited research that assesses the contribution of the family factors for the development of treatment modalities and strategies to thwart suicide.

Although suicide is a public health problem in Bangladesh, prevention has received little attention, evidenced by no central suicide prevention strategy (Arafat, 2018; Khan et al., 2020). Suicide is still considered a criminal offense in the country (section 309 of the Penal Code 1860) along with cultural stigma and social myths associated with suicide and low suicide literacy (Arafat et al., 2022; Khan et al., 2020; United for Global Mental Health, 2021). Due to this, people are fearful of sharing suicide intent, seeking help, admitting previous suicide attempt or suicide completion in family by a member to avoid social or legal harassments. The threat of punishment almost does not prevent an individual who take his own life by completing suicide (United for Global Mental Health, 2021). Sporadic prevention strategies have been started in Bangladesh without any harmonization despite the fact that the WHO has been asserting the necessity of governmental involvement (Arafat, 2019). Risk factors for suicide in Bangladesh have not been studied extensively, nor has the role of the family in suicide prevention. We aim to highlight the areas of family vulnerability and resilience when the threat of suicide is present, as well as the potential roles of family in suicide prevention in Bangladesh.

## 2 METHODS

We conducted a thorough literature search and narratively synthesized evidence based on available articles discussing suicidality and family dynamics in Bangladesh. Initially, we identified articles discussing suicidality in Bangladesh. The search was conducted in PubMed, Scopus, and Google Scholar with the search term “suicide in Bangladesh.” We included articles published in the English language from inception to 2020. We also performed a hand search of three available review articles (two systematic reviews and one narrative review) on suicide in Bangladesh (Arafat, 2017, 2019; Arafat et al., 2021). We scrutinized for risk factors for suicide related to the family from the articles and narratively synthesized evidence. We also discussed the family structure and family dynamics from Minuchin's structural model of family systems. We proposed possible prevention strategies based on the conception formulated by the narrative synthesis.

## 3 RESULTS AND DISCUSSION

### 3.1 Family as an interactive system

Minuchin's (1974, 1982) structural model of family systems identifies three primary subsystems: spousal subsystem, sibling subsystem, and parent–child subsystem. The spousal subsystem is of particular interest because it links three generations: the marriage, their children, and each spouse's family of origin. Any expected and effective change in children is often facilitated through a change in the spousal subsystem, and change in the spousal subsystem commonly comes from addressing issues with their parents and other family members.

The subsystems are separated by internal boundaries, which regulate closeness and distance while determining membership. For example, the spousal subsystem ideally includes the parents only, but can sometimes include children when the children have been parentified. External boundaries define the relationship between the family and larger systems, such as education and health systems. When assessing boundaries, clinicians determine the permeability of boundaries in several forms such as impermeable/closed, semi‐permeable/open, and permeable/diffuse. Semi‐permeable boundaries allow for a balance of individual autonomy and closeness between family members or between the family and larger systems in the community.

Assessment of family structure is facilitated by an assessment of a family's flexibility and cohesion. Flexibility refers to the family's ability to adapt to change, which is intimately related to the family's leadership and organization. It has two primary ingredients. First, how does the family mold and reshape its structure to accommodate change? Some families will make the necessary adjustments in roles, responsibilities, and expectations to cope effectively with change, while others will resist such change. For example, a mother is diagnosed with a debilitating chronic illness and is unable to perform the functions normally expected of her. If the family fails to restructure their roles and responsibilities and pretends as if nothing has changed, it is highly likely that stress will increase and the family's functioning will suffer. The second ingredient of flexibility is the continuity that is measured by how is stability maintained in the face of change in a family? When a family is in the midst of change, they are ideally preserving some familiarity, such as daily routines and rituals. Achieving healthy flexibility means striking a delicate balance between continuity and change.

Cohesion refers to connection and support in families—what is the amount of caring, closeness, and affection in the family (Olson & Gorall, 2003)? Family members need to feel secure and safe in their environment, particularly when adversity and stress are high. During times of crisis, family members are ideally turning toward one another to listen and share concerns and offer any assistance that facilitates healing and recovery. When cohesion is lacking (an indication of disengagement), family members may feel disconnected and isolated and look outside the family for needed support. Although high cohesion is usually a strength in families, too much cohesion as evidenced by diffuse or weak internal boundaries (an indication of enmeshment) can block family members’ efforts to express individual preferences and may threaten physical and/or emotional privacy. Achieving healthy cohesion means striking a delicate balance between closeness and distance (Olson, 2000; Olson et al., 2007; Sanders & Bell, 2011).

Extreme imbalances in cohesion and flexibility have been linked to suicidal ideation and behavior (Berryhill et al., 2018; Gouveia‐Pereira et al., 2014; Wedig & Nock, 2007). For example, research has shown that adolescents from chaotic (low cohesion and flexibility) families have higher suicidal ideation than adolescents from a balanced family (Gouveia‐Pereira et al., 2014). Cohesion and flexibility have shown to a have protective role on adolescents' suicidal behavior (Gouveia‐Pereira et al., 2014). In addition, a lack of warmth and low perceived support, hostile or critical parenting strategies, poor communication, and lack of affection are also seen (Hilt et al., 2008; Tschan et al., 2015). In summary, familial factors and a lack of coping strategies can be contributors to suicidal behaviors (Berryhill et al., 2018; Liang et al., 2020).

Family structure is a culturally constructed basic unit of society. Theories of healthy family functioning have mostly been defined by the assessment of families in Western culture. What might be defined as healthy flexibility and cohesion in a white, middle‐class, American family may not be a healthy balance in families in other parts of the world. Next, we turn to a description of family structure in Bangladeshi culture.

### 3.2 Bangladeshi family structure

A family is defined as a group of people who are connected to each other by marriage, blood, or adoption, establishing a single unit, having interactions and communications with each other, and generating and preserving a mutual culture (De Silva, 2005). In South Asia, it is largely ruled by a patriarchal lineage. However, modernization is bringing change. For example, more women are entering the workforce, and there is a rise in youth empowerment that is influencing the experiences of new intimacies and companionship. Some changes are being observed, but the family institution is promulgating some normative behaviors and values (Bhandari & Titzmann, 2017). Other trends are also emerging. For instance, fertility rates are decreasing, and age at the time of marriage is increasing. While adolescent and arranged marriages continue to exist in South Asia, most elderly family members either continue to live with their adult children. Divorce and out‐of‐wedlock pregnancies continue to be relatively rare (Yeung et al., 2018).

In Bangladesh, family life and structure are changing. The average household size decreased from 5.7 in 1981 to 4.2 in 2019 (Bangladesh Bureau of Statistics, 2019). The number of households with five or fewer members has increased, and the number of households with 6 to 10 persons has decreased between 1991 and 2011 (Bangladesh Bureau of Statistics, 2015). A change in the household head has also been reported; female headed households have increased from 12.8% in 1997 to 14.6% in 2019 (Bangladesh Bureau of Statistics, 2019). Although separation and divorce continue to be uncommon in Bangladesh (National Institute of Population Research and Training et al., 2016), the divorce rate has increased from .47 (males) and 1.59 (females) to 2.7 between 2003 and 2019 (Bangladesh Bureau of Statistics, 2019).

The prevalence of domestic violence experienced by women in Bangladesh is 35% and household food insecurity is 86%. Age at marriage, lack of any support from household members, number of children, and decision‐making power of women at the household are significantly related to domestic violence (Haque et al., 2020). A range of negative outcomes are connected with inter‐partner violence, encompassing physical and psychological outcomes like chronic pain, respiratory conditions, perinatal problems, sexually transmitted infections, HIV, depression, anxiety, post‐traumatic stress disorder, drug abuse, and suicide (Beydoun et al., 2012; Dillon et al., 2013; Dokkedahl et al., 2019).

Bangladeshi families prefer high intimacy, cohesion, and interdependence among family members. Parents are supposed to take care of their children until they get married or sometimes even after marriage if the children are not financially stable. Similarly, the children take care of their parents, whether living in the same house or apart. Ideally, children follow the instructions, commands, and fulfill their expectations mostly without any argument. Children tend to see parental control as a reflection of parental warmth (Chowdhury & Rojas‐Lizana, 2020). Bangladeshi families prefer to retain their culture with the goal to keep their family bonding strong. Bangladeshi culture and family systems are mostly centered on family cohesion, with an extensive range of collectivistic family traditions (Chowdhury & Rojas‐Lizana, 2020).

Like other South Asian countries, Bangladeshi families sometimes, face conflicts when parents try to keep their children under the norms of their home culture (Ghuman, 2000). Research also indicates that Bangladeshi families have some flexibility to embrace new circumstances in order to help their children. They are found to be flexible in changing some of their culture‐related behaviors and practices that may not be consistent with their core values, such as close ties with extended family, patriarchal cultural values, religious observance, and marriage arrangements (Hughes et al., 2006).

### 3.3 Suicide risk factors in Bangladeshi families

Familial, social, physical, and psychological factors can contribute to and potentially exacerbate suicidal behaviors in Bangladesh. Identification of risk factors for suicide has been understudied in the country. A well‐designed case–control psychological autopsy study identified psychiatric disorders, life events, self‐harm, sexual violence, unemployment, and social isolation as the risk factors for suicide in an urban setting (Arafat et al., 2021). In the study, 61% had at least one mental disorder and 91% had exposure to life events. As per the Paykel's Life Events Schedule, the life events of 21% of suicides were closely associated with proximal factors related to family members such as arguments with a resident family member (10%), arguments with spouse (8%), marital separation associated with an argument (2%), and an argument with a non‐resident family member (1%) (Arafat et al., 2021). Another 47% of suicides were linked with life events indirectly related to family mentioning, such as an academic failure (9%), broken engagement (9%), sexual harassment (8%), financial problems (8%), death of a spouse (3%), business failure (2%), loss of job (2%), physical disease (2%), divorce (1%), a lawsuit (1%), forceful child marriage (1%), and marriage (1%) (Arafat et al., 2021). Relationship problems between the spouses were significantly higher among the cases compared with the controls (Arafat et al., 2021).

In 2007, nine members of a family died by suicide in a small town of Bangladesh; a suicide note was left (Selim, 2010). Extrapolation of the suicidal note revealed that the family members had shared delusion related to their religion. A community‐based study from rural Bangladesh revealed that the risk factors for approximately 63% of suicides were attributed to family problems (Feroz et al., 2012). Another case–control study identified that the risk factors for about 65.5% of suicides were attributed to family conflict (Reza et al., 2013). A narrative review found that the most common proximal deciding factors are related to the family, such as marital discord and conflict with family members (Arafat, 2017). Another scoping review reported that emotional distress due to family was the most common risk factor (Shahnaz et al., 2017). An empirical study assessed the media reports of suicide and revealed that about two‐thirds of the proximal factors were related to family issues, such as marital disharmony and conflict with family members (Shah et al., 2017). A systematic review of the literature identified several relational factors associated with suicidal behaviors, including sexual abuse, extramarital relationships, child marriage, death of partner and/or children, domestic violence, and divorce; marital discord and family conflict were the primary factors (Arafat, 2019).

**FIGURE 1:**
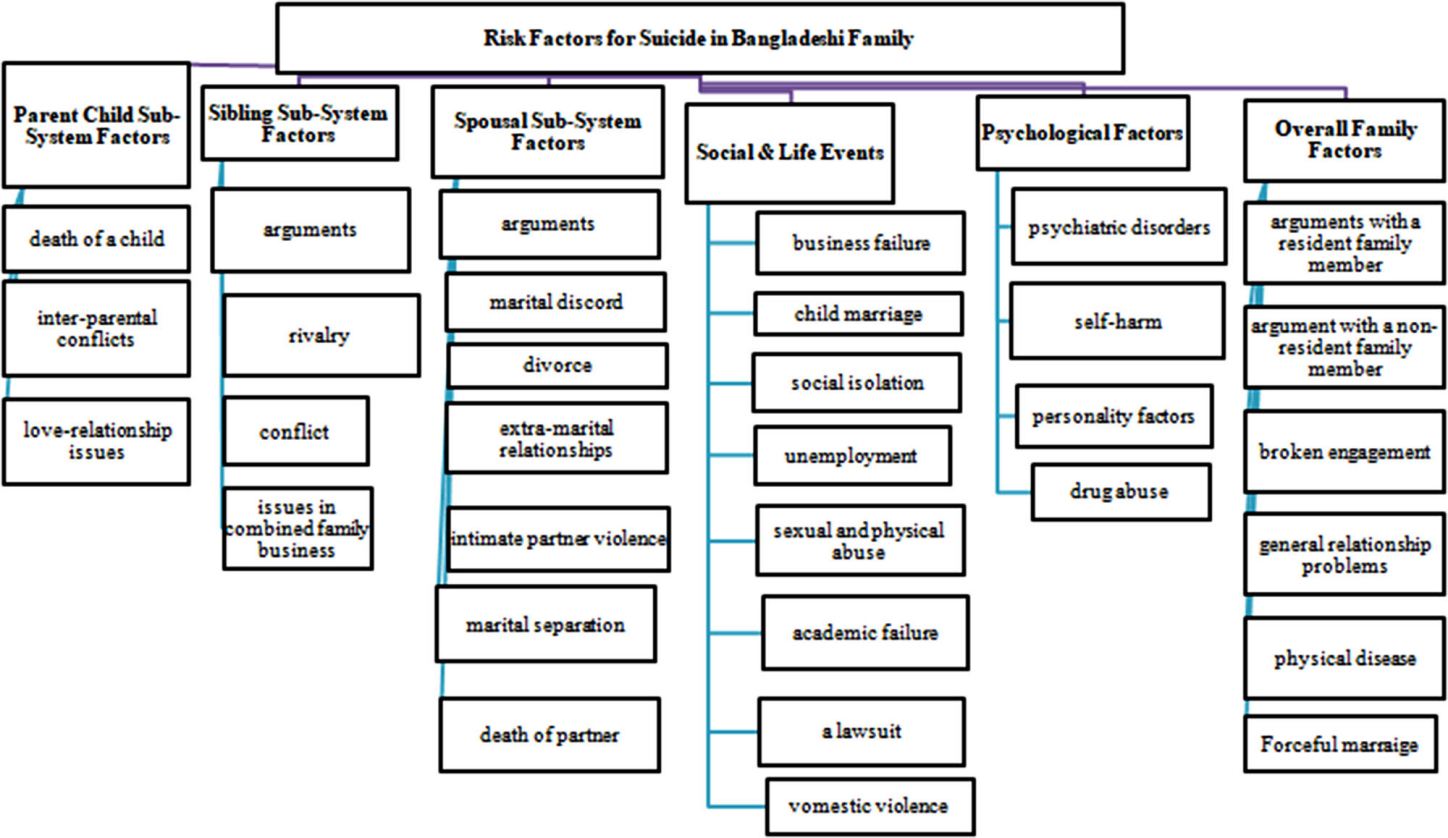
Risk factors for suicide in Bangladesh in different subsystems of family

In the spousal subsystem, chronic conflict, marital separation, divorce, forced marriages, and general relationship problems are considered to be predictors of suicide in Bangladesh (Figure 1). Similarly, some factors related to the parent–child subsystem are forceful child marriage, academic failure, death of children, abuse in school, love–relationship issues, domestic violence, and authoritarian parenting. In the sibling subsystem, there may be a role of broken engagements, financial issues, issues in combined family business or business failure, and arguments. There has been recognition of sibling rivalry, and it is observed that children learn to compete for attention, the fulfillment of needs, and empowerment within their families. Sometimes, there may be unresolved rivalries and non‐supportive or strained relationships among the siblings that may lead to suicide.

Bangladesh has been experiencing an increase in domestic violence (Islam et al., 2014). Research has shown that suicidal ideation was more common among abused women in Bangladesh (Johnston & Naved, 2008; Naved & Akter, 2008). The authors recommended initiatives to curb domestic violence against women (Naved & Akter, 2008). The psychological autopsy study also revealed that 40% of the life events were closely related to inter‐personal violence associated with marital and sexual issues such as spousal discord, extramarital affair, forceful marriage, premarital love relationship, and quarrel with family members (Arafat et al., 2021).

### 3.4 Proposed family‐centric suicide prevention strategies in Bangladesh

**FIGURE 2:**
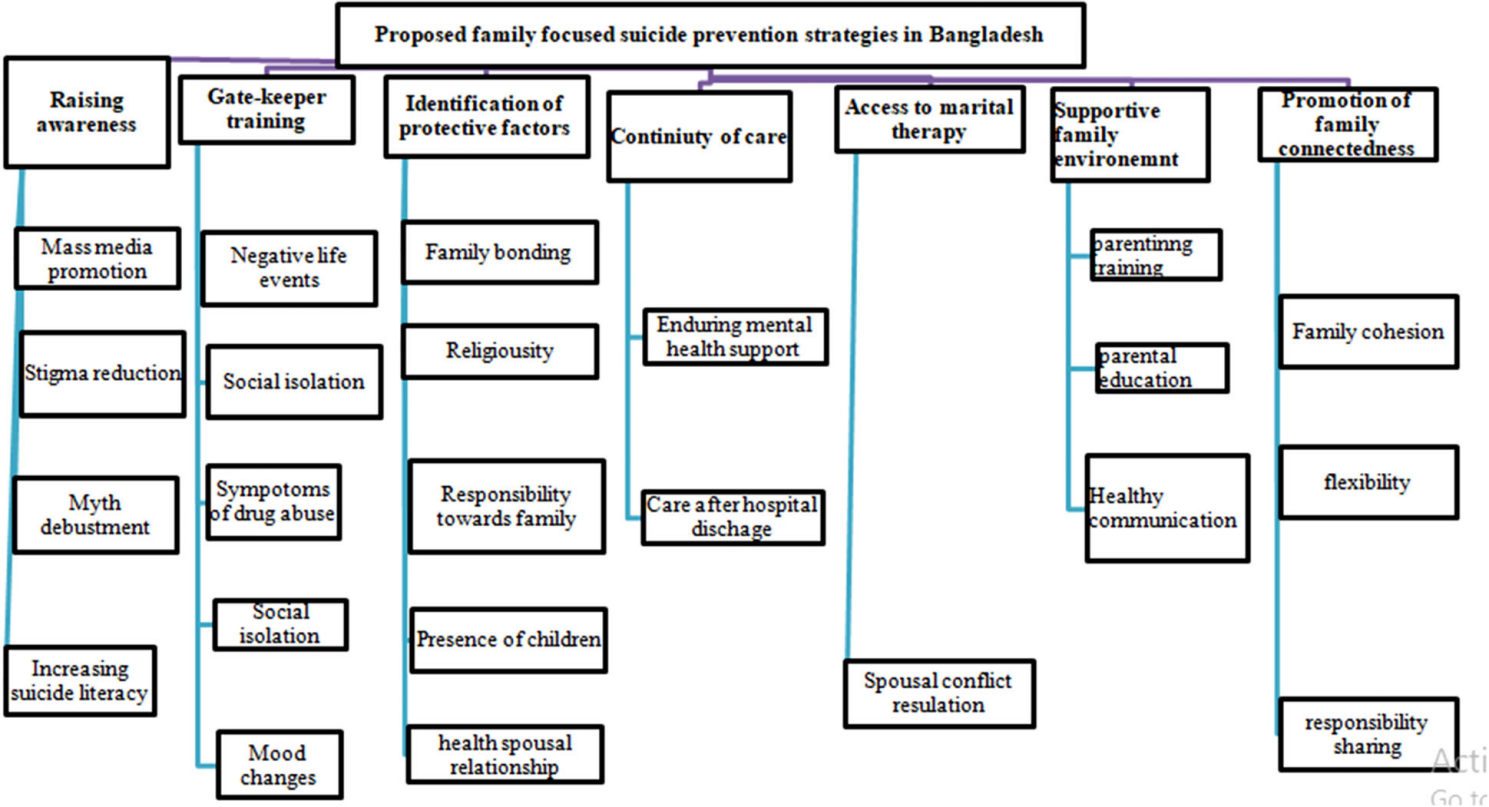
Proposed family‐focused suicide prevention strategies in Bangladesh

A supportive family environment, healthy relationships among the family members, and open communication may help prevent suicidal behaviors (Edwards et al., 2021). Unfortunately, individuals with suicide ideation often avoid the disclosure of suicidal thoughts to their family members. The WHO advocates the involvement of family members while formulating the management plans of psychiatric patients, especially in low‐ and middle‐income countries due to low resources and weak national mental health services (Edwards et al., 2021). However, it has not been applied and tested in Bangladesh. We suggest the prevention strategies to increase suicidal behavior and increase family resilience (Figure 2).

#### 3.4.1 Raise awareness

Raising awareness among family members, as well as the general population through mass media, should be considered as a potential prevention strategy. Dispelling myths among the family must be prioritized. Several myths should be targeted such as “suicidal persons do not express the intent,” “persons who express the suicidal intent usually do not attempt,” and “enquiring about suicidality increases suicide” (Edwards et al., 2021). It has been identified from previous evidence that people used to hide the suicidal intent, behavior, and incidence in Bangladesh due to stigma and legal harassments.

#### 3.4.2 Gatekeeper training

Family members can be trained as gatekeepers for the identification of at‐risk members of a family. To date, gatekeeper training has not been started in Bangladesh, and also no community mental health services team has been formulated in the country (Arafat, 2019). The family leaders could be trained to identify risky symptoms, such as recent mood changes, any sign of substance abuse, social isolation, and negative life events (Arafat & Kabir, 2017). They should also be informed about available preventive services.

#### 3.4.3 Identification of protective factors

Attention to protective factors related to the family should be considered. Religious beliefs and the presence of young children are key protective factors for suicidal attempts in Bangladesh. Several factors have been recommended to consider while identifying the protective factors, such as the purpose of living, responsibilities towards other family members, exploring the core of oneself, and emotion regulation (Edwards et al., 2021).

#### 3.4.4 Continuity of care

Family members can be a link between the mental health support team and patient, particularly after discharge from the hospital, which can be a risky and frightening time (Arafat & Kabir, 2017; Edwards et al., 2021; Zalsman et al., 2016). Such continuity is a challenge in Bangladesh due to poor suicide literacy, a high mental health treatment gap, and stigma related to suicide (Arafat et al., 2022; Arafat, 2019).

#### 3.4.5 Increase access to marital therapy

Programs are also needed to assist spouses in managing their conflicts. A healthy marriage is vital to the overall health of the family. The spousal subsystem has an influence on the other subsystems and has the power to increase or decrease vulnerability.

#### 3.4.6 Supportive family environment and appropriate parenting

The first peer relationship is between siblings. Attention should be paid to sibling conflicts in the early years. Parents need to intervene when children disagree in order to help them solve problems. When left unresolved, sibling conflicts may later become violent and abusive (Arafa et al., 2021; Button & Gealt, 2010; Feinberg et al., 2012). Parental strategies for managing such arguments, such as negotiation and fair resolutions, could be significant for learning how to get along with others. However, parental education and sibling intervention programs are needed.

#### 3.4.7 Promote family communication and connectedness

Effective communication and supportive family relationships can help safeguard a family member against suicide, regardless of the existence of other risk factors. Promotion of family cohesion, flexibility, expressiveness could protect from developing depression, hopelessness, anxiety, and suicide behavior (Ahookhosh et al., 2017; Gouveia‐Pereira et al., 2014).

## 4 CONCLUSION

The family has an important role to play in suicide prevention in Bangladesh. To date, the potential prevention strategies and their effectiveness have been untapped in the country. Studies are warranted to test the effectiveness of the proposed strategies. Policy makers should include family education and support services while formulating national suicide prevention strategies. Mental health professionals should consider the potential vulnerable and protective factors existing within the family during the assessment of suicidal intent as well as during the management of non‐fatal attempts. The mental health professionals could develop family‐focused interventions in order to enhance family functioning, resolve interpersonal issues, increase family integration, and social support. Such programs may be developed, in which families may be involved in the prevention and treatment of suicide risk. This also calls out for trainers, academicians, and educational institutions in developing and implementing such programs. Mass media could highlight the family connectedness, responsibility, and identity of self while reporting the suicidal behaviors.

## CONFLICT OF INTEREST

The author(s) declared no potential conflicts of interest with respect to the research, authorship and/or publication of this article.

## AUTHOR CONTRIBUTIONS

*Conceptualization*: S. M. Yasir Arafat, Murad Khan. *Writing – original draft*: S. M. Yasir Arafat and Tamkeen Saleem. *Writing – review and editing*: All authors. *Final approval*: All authors.

### PEER REVIEW

The peer review history for this article is available at https://publons.com/publon/10.1002/brb3.2562

## Data Availability

Data sharing is not applicable to this article as no new data were created or analyzed in this study.
